# Supplementary material for: PXL: a Nucleic Acid–Binding Module of Promyelocytic Leukemia Protein
Source: bioRxiv. 2026 Feb 20:2026.02.19.706916. Preprint. [Version 1] doi: 10.64898/2026.02.19.706916 (PMC12934764; doi:10.64898/2026.02.19.706916)
Supplement: Supplement 1 [file media-1.pdf]

PXL: A Nucleic Acid–Binding Module of Promyelocytic Leukemia Protein

Daniel Fairchild, Irina V. Semenova, Dane Geddes-Buehre, Yunfeng Li, Renata Szczepaniak, Sandra K. Weller, Bing Hao, Irina Bezsonova*

Department of Molecular Biology and Biophysics, School of Medicine, University of Connecticut, 263 Farmington Avenue, Farmington, CT, 06030, United States

*Correspondence to bezsonova@uchc.edu

Supplementary Information

Supplementary figure 1

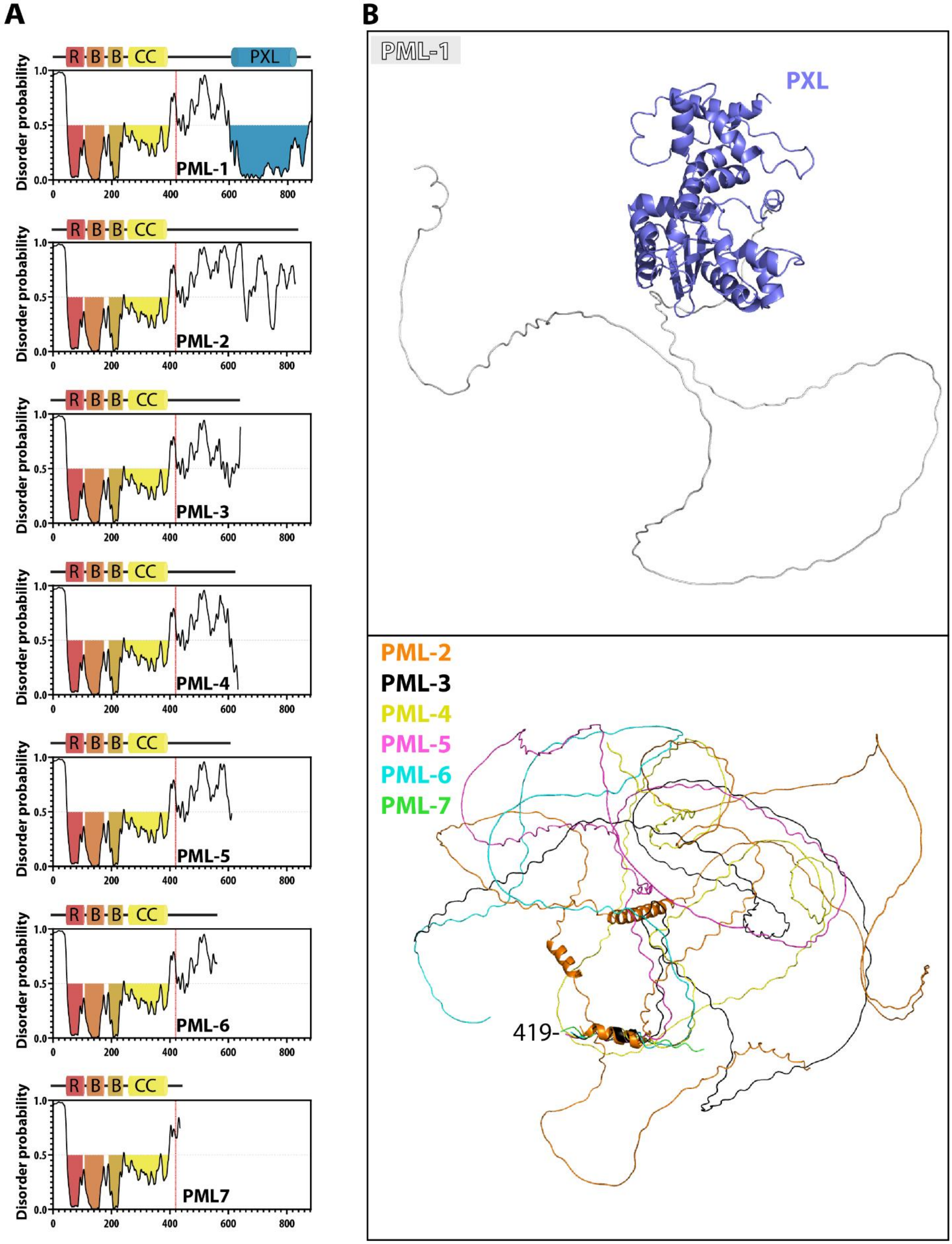

Supplementary Figure 1: The unique C-terminus of PML-1 is predicted to contain an ordered domain. (A) IUpred3 disorder predictions of all full length PML isoforms. Isoform specific C-termini begin at residue number 419 (vertical red line). PML-1 is predicted to contain an ordered region in its unique C-terminus. **(B)** AlphaFold3 predictions of PML-1 C-terminus (top) and PML-(2-7) (bottom).

Supplementary figure 2

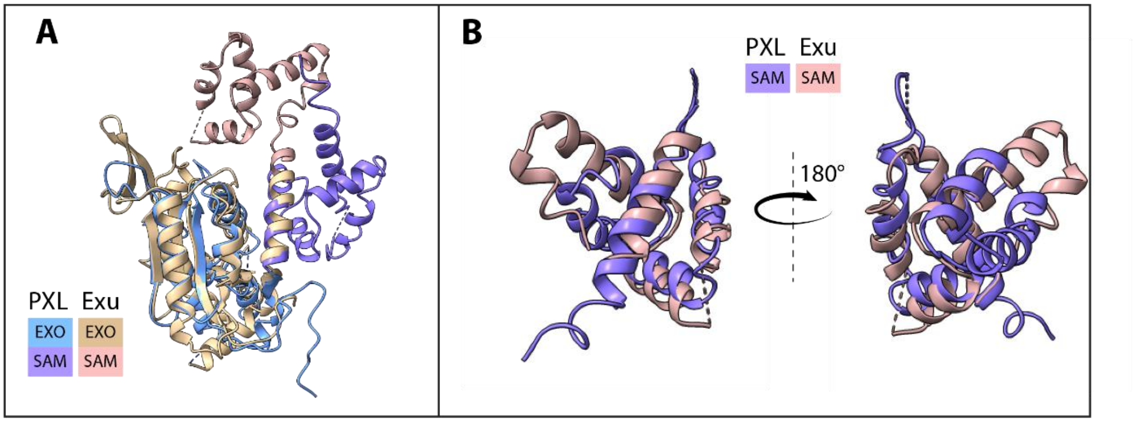

Supplementary Figure 2: PXL and Exu SAM-like domains share secondary structure but differ in orientation relative to the Exo-like domain.

(A) Overlay of the PXL and Exu exo-like (PXL: blue, Exu:Tan) and SAM-like (PXL: purple, Exu:pink) domains. The SAM-like domains differ in their orientation with respect to their Exo-like domains. **(B)** Overlay of the PXL SAM-like (purple) and Exu SAM-like (pink) domains showing conserved secondary structure alignment.

Supplementary figure 3

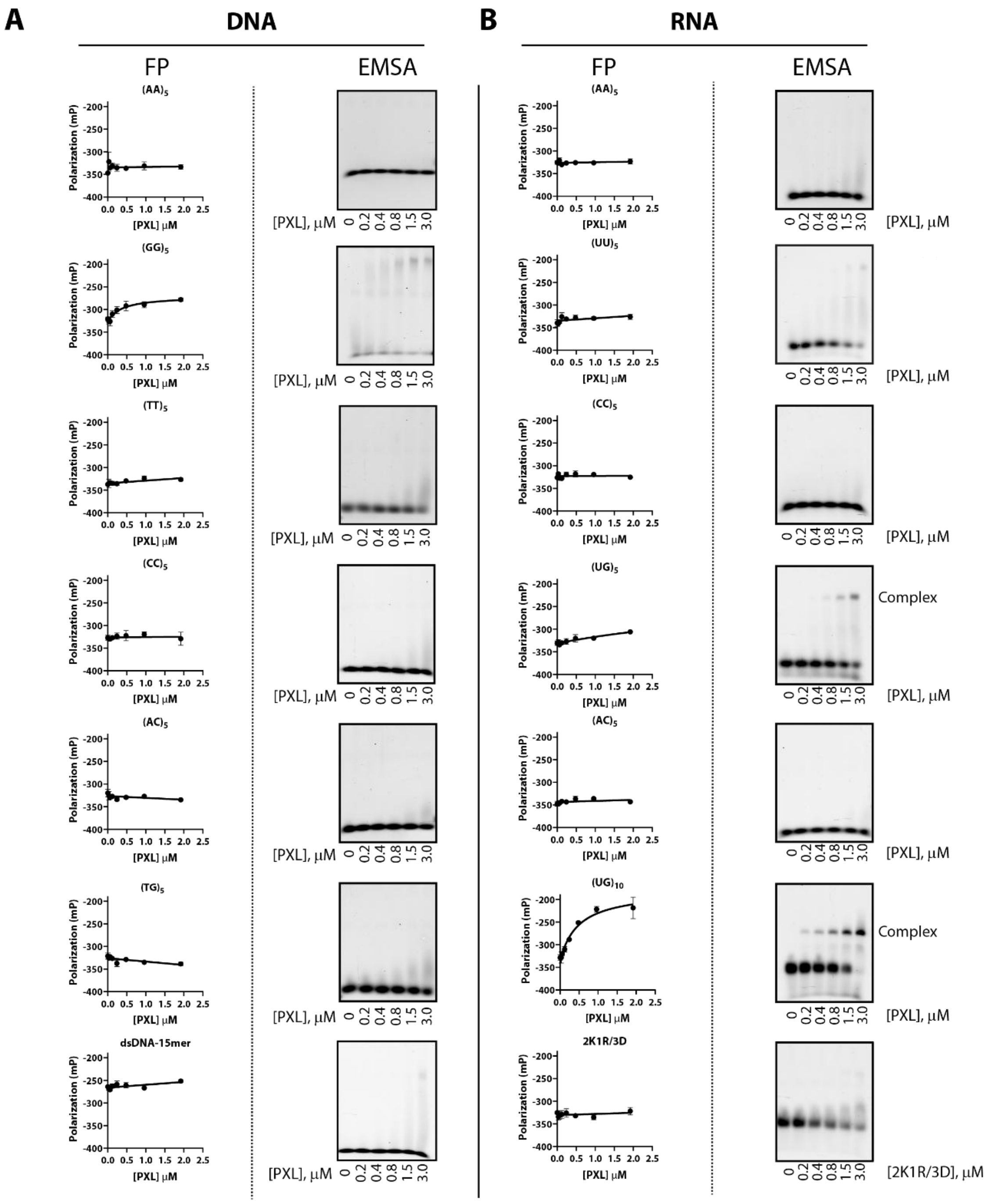

Supplementary Figure 3: PXL selectively binds poly(UG) RNA sequences utilizing positively charged residues on both the SAM-like and Exo-like domains.

(A) Fluorescence polarization (left) and electrophoretic mobility shift assays (right) of all DNA constructs used to assess PXL binding. **(B)** Fluorescence polarization (left) and electrophoretic mobility shift assays (right) of all RNA constructs used to assess PXL binding and the PXL 2K1R/3D binding deficient mutant.

Supplementary figure 4

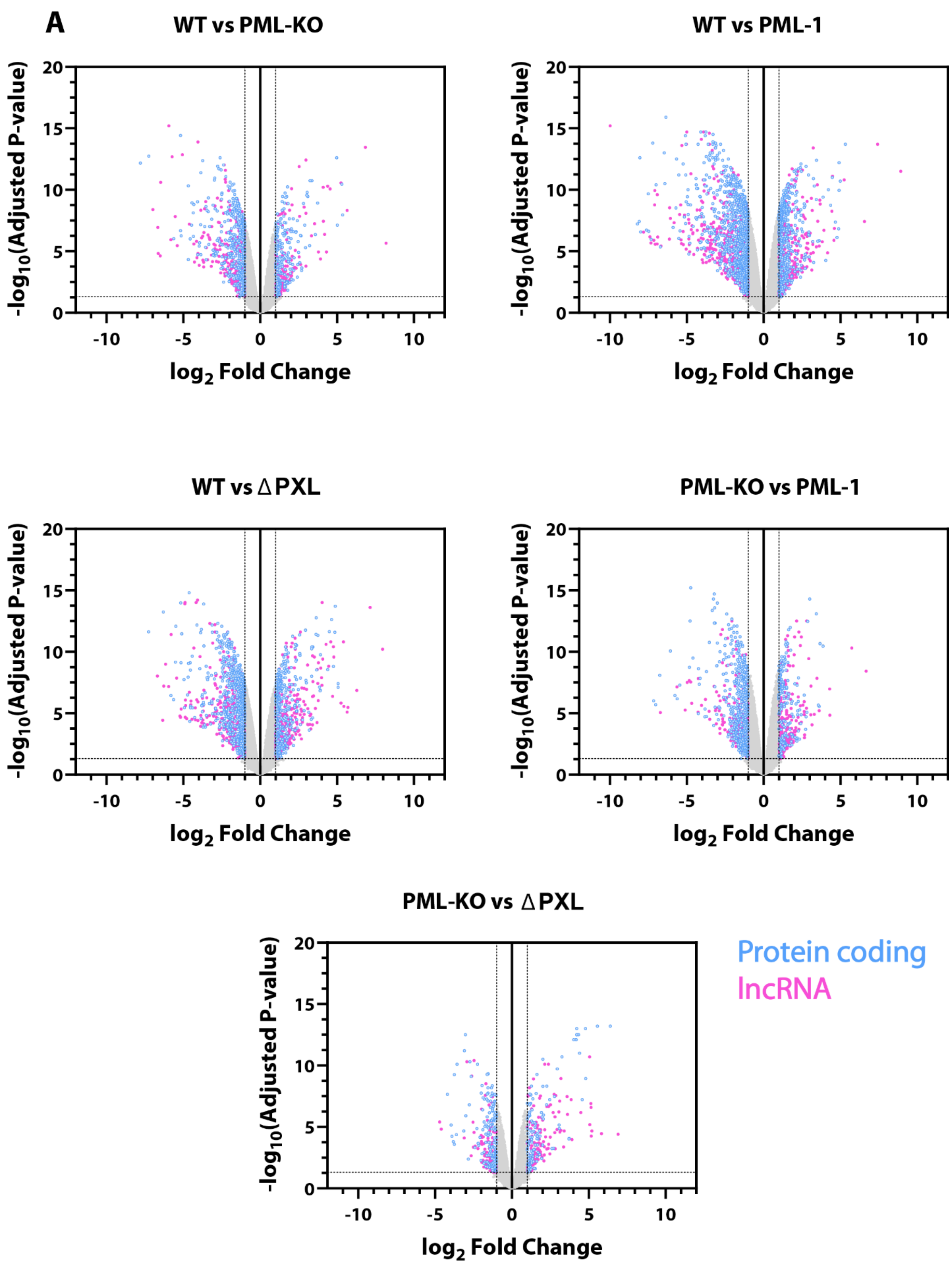

Supplementary Figure 4: The PXL domain of PML-1 affects the transcriptome of U2OS cells.

(A) Volcano plots comparing changes in transcriptomes across U2OS cell lines showing significant changes in the abundance of both lncRNAs (pink) and protein coding genes (blue).
